# Relationships between Translation and Transcription Processes during fMRI Connectivity Scanning and Coded Translation and Transcription in Writing Products after Scanning in Children with and without Transcription Disabilities

**DOI:** 10.4236/ce.2017.85055

**Published:** 2017-04-30

**Authors:** Peter Wallis, Todd Richards, Peter Boord, Robert Abbott, Virginia Berninger

**Affiliations:** 1 Educational Psychology—Learning Sciences and Human Development, University of Washington, Seattle, USA; 2 Integrated Brain Imaging Center, Department of Radiology, University of Washington, Seattle, USA; 3 Educational Psychology—Quantitative Studies, Statistics and Measurement, University of Washington, Seattle, USA

**Keywords:** Transcription, Translation, Dysgraphia, Dyslexia, Typical Writing

## Abstract

Students with transcription disabilities (dysgraphia/impaired handwriting, *n* = 13 or dyslexia/impaired word spelling, *n* = 16) or without transcription disabilities (controls) completed transcription and translation (idea generating, planning, and creating) writing tasks during fMRI connectivity scanning and compositions after scanning, which were coded for transcription and translation variables. Compositions in both groups showed diversity in genre beyond usual narrative-expository distinction; groups differed in coded transcription but not translation variables. For the control group specific transcription or translation tasks during scanning correlated with corresponding coded transcription or translation skills in composition, but connectivity during scanning was not correlated with coded handwriting during composing in dysgraphia group and connectivity during translating was not correlated with any coded variable during composing in dyslexia group. Results are discussed in reference to the trend in neuroscience to use connectivity from relevant seed points while performing tasks and trends in education to recognize the generativity (creativity) of composing at both the genre and syntax levels.

## 1. Introduction

Of the brain imaging studies of individuals with specific learning disabilities (SLDs), relatively more of these studies have focused on reading disabilities than writing disabilities. Yet research around the world has shown that writing disabilities (a) co-occur in individuals with reading disabilities and oral language disabilities or may occur in the absence of reading and oral language disabilities (Arfé, Dockrell, & Berninger, 2015; Berninger, 2015); and (b) occur in a sizable percent of the school-age population alone or with other SLDs, but have been the forgotten SLD that has been left behind (Colligan & Katusic, 2015).

Brain imaging studies of writing disabilities that have been done focus on either developmental writing disorders in which a writing skill does not develop in a typical way or acquired writing disorders in which a previously acquired writing skill is lost, typically through injury or disease. Imaging studies of the writing brain have used a variety of imaging tools to study motor processes (e.g., Katanoda, Yoshikawa, & Sugishita, 2001), handwritten letter production (e.g., James & Engelhardt, 2012; Longcamp et al., 2014; Longcamp, Richards, Velay, & Berninger, 2017; Richards, Berninger, Stock, Altemeier, Trivedi, & Maravilla, 2011), spelling (Booth, Cho, Burman, & Bitan, 2007), or cognitive processes (Berninger et al., 2009; Shah, Erhard, Ortheil, Kaza, Kessler, & Lotze, 2013). Cognitive writing researchers refer to handwriting and spelling as the transcription skills the scribe uses to record the translated cognitions in written language, but distinguish between transcription—the recording of written language—and translation—the transformation of thought into language (Hayes, 1996; Fayol, Alamargot, & Berninger, 2012). The translation process involves creativity (idea generation), planning for transforming those ideas into written language, and the cognitive linguistic transformation process itself.

More imaging studies have focused on transcription because it is easier to study transcription than translation within the scanning environment. However, Shah et al. (2013) contrasted planning and creative writing (translation processes) and copying (transcription process) in adults using a subtraction paradigm. Likewise, the current study included both translation and transcription processes but contrasted children who did or did not have a specific developmental writing disability in transcription (either subword handwriting or word spelling) in the relationships between specific transcription or translation tasks during connectivity scanning with coded transcription or translation variables in composition products outside the scanner.

The current study had three research aims. The first research aim was to develop a coding scheme for the compositions planned during scanning but written outside the scanner for both the transcription and translation variables observed in the writing outcomes at the behavioral level. This first step for analyses at the behavioral level was a necessary first step for examining the brain-behavior relationships between transcription and translation processes during scanning and the writing products outside the scanner.

The second research aim was to evaluate whether the three diagnostic groups, who were identified based on comprehensive assessment with normed measures prior to brain scanning as having or not having dysgraphia or dyslexia, differed on the coded transcription outcomes in the compositions written outside the scanner. At issue was whether the three diagnostic groups that had differed on normed measures also differed on writing products during authentic composing activities involving both translation and transcription. The first hypothesis tested was that the three groups would differ significantly on the coded transcription and translation variables in the compositions written outside the scanner.

The third research aim was to evaluate (a) whether fMRI connectivity during brain scanning on transcription tasks was correlated with coded transcription variables in composing after scanning, and (b) whether fMRI connectivity during brain scanning on translation tasks was correlated with coded translation variables in composing after scanning. Of interest was whether such correlations, if significant, varied in patterning across the diagnostic groups. The second tested hypothesis was that for all diagnostic groups the transcription tasks during scanning would correlate with coded transcription variables in compositions outside the scanner and the translation tasks during scanning would correlate with coded translation variables in compositions outside the scanner.

To achieve these three research aims and test two hypotheses, children who had met research criteria for one of the three diagnostic groups then completed four tasks during fMRI connectivity scanning—two cognitive ones related to translation (first and last) and two transcription ones (second and third). The first was resting condition shown in imaging research to be associated with self-guided mind wandering (Raichle, MacLeod, Snyder, Powers, Gusnard, & Shulman, 2001), which may contribute to flow of thoughts during writing (Kellogg, 1994) and creative writing (Shah et al., 2013). The second was a handwriting task—write the letter in the alphabet that comes after the letter visually displayed on the screen. The third was a spelling task—add a letter to the blank in the visually displayed word to create a correctly spelled real word. The fourth was planning a composition for a topic announced at the end of the scan while the child’s brain was scanned. The composition was written immediately after children got out of the scanner and later was coded by the research team for translation and transcription variables.

An interdisciplinary approach to data analysis was employed. The analysis of the brain data was informed by and analyzed using a connectionist framework (Sporns, 2013). Magnitude of connectivity from four seed points was used: precuneus, which human connectome brain research has shown is in the rich club of neural networks and may participate in multiple networks in the human connectome (van den Heuvel & Sporns, 2011) including those supporting planning and creative expression during translation (Shah et al., 2013); and three identified in meta-analyses of brain imaging studies for written words that are relevant to spelling and reading written words—left occipital temporal, supramarginal gyrus, and inferior frontal gyrus (Purcell, Turkeltaub, Eden, & Rapp, 2011). The coding of the compositions outside the scanner used linguistic coding of transcription and translation variables informed by current cognitive research in writing (Hayes, 1996; Fayol et al., 2012). Two coders developed and applied the coding scheme and established interrater reliability for using it.

## 2. Method

### 2.1. Participants

Children in grades 4 to 9 who were right handed and did not wear metal that could not be removed completed comprehensive differential diagnostic assessment. Normed measures and evidence-based guidelines validated in two decades of research (Berninger & Richards, 2010; Berninger, Richards, & Abbott, 2015) were used along with parent interviews and questionnaires to document past and current persisting problems in hand-writing or in spelling despite intervention or absence of past or current hand-writing or spelling problems and also to rule out other neurological or neurogenetic conditions other than specific learning disability that could account for handwriting or spelling problems.

Criteria for dysgraphia in addition to history of past and current handwriting struggles were test scores below −2/3 SD on two or more handwriting measures (writing alphabet from memory, copying a sentence with all letters of alphabet in one’s best handwriting, copying a sentence with all letters of alphabet in one’s fastest handwriting) (Berninger et al., 2015). Criteria for dyslexia, which is a word spelling disability and not just a word reading disability (Bruck, 1993; Connelly, Campbell, MacLean, & Barnes, 2006; Lefly & Pennington, 1991), was the same as used in an 11-year family genetics study (Berninger & Richards, 2010) and study of persisting specific learning disabilities during middle childhood and adolescence (Berninger et al., 2015). In addition to past and current history of struggles in word reading and spelling, criteria for impaired accuracy or rate of oral real word reading and/or decoding or silent word reading fluency and for impaired spelling (dictated real word spelling, dictated pseudo word spelling, identifying the correct word-specific spelling among homophone foils, unscrambling anagrams to create a word-specific spelling, and/or adding a missing letter to create a word-specific spelling) had to be met on two or more measures: below the population mean and at least one standard deviation below Verbal Comprehension Index. Most met criteria on more than two measures and were more impaired than the minimum criteria required. All in the current study were impaired in word spelling.

Altogether 39 had participated. Of these, 14 (11 males and 3 females, age in months, M = 143.64, SD = 15.97) met research criteria for dysgraphia, 16 (9 males and 7 females, age in months, M = 144.31, SD = 19.42) met research criteria for dyslexia, and 9 (4 males and 5 females, age in months, M = 159.33, SD = 15.59) met research criteria for controls (no evidence of dysgraphia or dyslexia). The ethnicities of participants in the sample were representative of the area in which it was conducted, with the majority European American (*n* = 31), but also Asian American (2), Hispanic (2), Middle Eastern (1), Other (1), or missing (2). Highest parental level of education ranged from high school (2 mothers, 2 fathers) to more than high school but less than college (1 mother, 2 fathers) to college (16 mothers, 15 fathers) to more than college (17 mothers. 16 fathers); level of education was missing for 3 mothers and 4 fathers.

### 2.2. Procedures

#### Tasks during scanning

Each participant received training outside the scanner and completed four tasks in this order in the scanner: 1) no experimenter-defined task (RESTING STATE), 2) production of the letter that follows a visually displayed letter in alphabet order (ALPHABET WRITING task), 3) production of letter in the blank in a visually displayed letter string to create a correctly spelled word (SPELLING WRITING task), and 4) planning a composition on an experimenter-provided topic (PLANNING task). During the fMRI writing tasks, a mirror system enabled the participant in the scanner to see the instructions and task on a screen. The tasks and writing pad recordings were all programmed, timed, and coordinated with the scanner triggers using E-prime and in-house LabView software.

The resting condition lasted for 6 min 14 s. It was followed by 6 s of instruction for the alphabet task. The alphabet writing task lasted for 4 min and was self-paced. After the visual display of the first letter, the child wrote the next letter in the alphabet. When the child lifted the pen off the tablet, visual display 2 appeared and the process repeated for 4 min. Next there were 6 s of instruction for spelling followed by the spelling task that lasted for 4 min and was self-paced. After visual display 1, the child wrote a letter in the blank to complete the word spelling. When the child lifted the pen off the tablet, visual display 2 appeared and the process repeated for 4 min. The response requirement was the same for the alphabet and spelling tasks—to form one letter using the MRI-compatible stylus. Next, instructions for planning stayed on the screen for the whole 4 min, while the child just generated ideas and planned a composition on a topic provided, but did no writing until leaving the scanner. Aural instructions for the last task were as follows: “*After you leave the scanner you will write about ‘Astronauts Writing While Exploring Outer Space’. Please start thinking in the inner space of your mind now about the ideas you will write about*. Identical instructions readable by the children lying prone in the scanner were also displayed.

#### Collecting compositions

When students got out of the scanner, they wrote their compositions with paper and pens. Scanner staff collected the compositions, scanned them to create pdf files, which were entered into a database for later retrieval for analysis.

#### Developing coding schemes for compositions and interrater reliability

The goal of the coding was to capture what was observed in participants’ compositions regarding handwriting quality and number of spelling errors (indictors of transcription), and number of words, staying on topic, text organization, creative expression, and genres (indicators of translation). The initial coding scheme for the compositions written outside the scanner was developed by two independent coders, who each inspected a subsample of ten written compositions and created a coding scheme for all that they observed. Then they met, discussed their coding, and refined the coding scheme by coding an additional 20 compositions and discussing any discrepancies until they were resolved. Finally, they independently applied the coding scheme to an additional seven compositions and reached 100% interrater agreement for coding each outcome, except for two items related to genre, which were discussed until agreement was achieved.

#### Imaging

Functional magnetic resonance imaging (fMRI) connectivity scans on a Philips 3T Achieva scanner (release 3.2.2 with the 32-channel head coil) were used to obtain measures of functional connectivity. The following fMRI series were scanned: fMRI scan with echo-planar gradient echo pulse sequence (single shot): TR/TE 2000/25 ms; Field of view 240 × 240 × 99 mm; slice orientation transverse, acquisition voxel size 3.0 × 3.08 × 3.0 mm; acquisition matrix 80 × 80 × 33; slice thickness 3.0, 13:08 min/s. Functional images were corrected for motion using FSL MCFLIRT (Jenkinson et al., 2002), and then high-pass filtered at sigma = 20.83. Motion scores (as given in the MCFLIRT report) were computed for each subject and average motion score (mean absolute displacement) for each of the groups: control 1.31 ± 1.37 mm, dysgraphic 1.50 ± 1.23 mm, and dyslexic 1.47 ± 1.03 mm. Spikes were identified and removed using the default parameters in AFNI’s 3d Despike. Slice-timing correction was applied with FSL’s slicetimer; and spatial smoothing was performed using a 3D Gaussian kernel with FWHM = 4.0 mm. Time series motion parameters and the mean signal for eroded (1 mm in 3D) masks of the lateral ventricles and white matter (derived from running Free Surfer 3s recon-all on the T1-weighted image) were analyzed.

Co-registration of functional images to the T1 image was performed using boundary based registration based on a white matter segmentation of the T1 image through epi_reg in FSL. The MPRAGE structural scan was segmented using FreeSurfer software; white matter regressors were used to remove unwanted physiological components. Group maps for fMRI functional connectivity were generated for the 3 groups (control, dysgraphia, and dyslexia) for each of the 4 tasks/conditions from 4 different seed points (in the left precuneus cortex PCC, in the left temporoocipital cortex TOC, in the left supramarginal gyrus SMG, or in the left inferior frontal gyrus, IFG Broca’s area with eight brain regions). These eight regions included four associated with written language processing (Broca’s, fusiform, supramarginal, and fusiform 2), one associated with executive functions (cingulate), one associated with attention (thalamus), one associated with working memory (hippocampus), and one associated with emotions such as fear and anxiety (amygdala). fMRI time-series were averaged within regions of interest (ROIs) formed from a 15 mm sphere centered at each seed. The averaged time-series at each ROI was correlated with every voxel throughout the brain to produce functional connectivity correlation maps, converted to z-statistics using the Fisher transformation.

Individual functional connectivity values from all four seed points were extracted from the coregistered connectivity maps from the following brain region of interests (ROI): 1) broca_IFG (70 67 39, x, y, z, voxel coordinates from 2 mm MNI space); 2) fusiform (29 38 24); 3) supramarginal_gyrus (69 42 46); 4) fusiform2 (59 22 29); 5) cingulate (47 61 57); 6) thalamus (51 57 41); 7) hippocampus (56 56 26); and 8) amygdala (57 62 26). In other words, for each participant on each task, functional connectivity values were obtained from each seed point with co-activated brain regions on each task, which were used in the statistical correlational analyses. Only functional connectivity magnitude values found to be statistically significant (using FSL’s randomise for group analysis, *which corrects for multiple voxels comparisons*, were correlated with coded data from the compositions written outside the scanner.

## 3. Results

### 3.1. Research Aim 1: Coding Transcription and Translation Skills during Composing Following Scanning

#### Coding transcription

The following scheme was used to code *handwriting quality*.

1. Most of the handwritten letters throughout the text are not legible (identifiable outside the context of a word and maintain relative proportionality and discriminating features in reference to other alphabet letters).
2. Reader has to work hard to decipher the handwriting throughout the text (using word context and other clues to figure out intended word).
3. In general the handwriting is legible and the reader only has to try to decipher it occasionally.
4. The handwriting is legible without reader having to work to decipher it throughout the text but does not stand out as the highest level quality of penmanship.
5. The handwriting is legible without reader having to work to decipher it throughout the text and stands out as high level quality of penmanship.

For *spelling errors*, the score was the total number of words that did not use conventional spelling for the word meaning in the sentence context.

#### Coding staying on topic during translation

Because topic is important in developing translation skills in composing during middle childhood (Olinghouse, Graham, & Gillespie, 2015), compositions were also analyzed according to whether they were about the researcher-provided topic, that is, contained content relevant to topic of *astronauts writing while exploring outer space*. This topic has a proposition—use of writing while astronauts explore space, but some students wrote selectively only about astronauts and space exploration, or diverged entirely from any topic in instructions. Some started out on the topic provided at the end of the scanning session, but then got off topic, whereas others never really addressed the stated topic. Compositions were coded for being on and staying on-topic, on a scale of 1 – 5, using the following scheme:

1. Participant only mentioned astronauts in passing, or not at all.
2. Participant wrote about astronauts but then got off on other topics.
3. Participant wrote primarily about astronauts, but not about their writing.
4. Participant wrote about astronauts and about the astronauts writing but did not link the two “while” exploring in outer space.
5. Participant wrote about astronauts writing while exploring outer space.

#### Coding text organization quality in translation

The follow coding scheme was used to code for text organization quality resulting from the translation process:

1. List.
2. Sentence preparing reader for next item(s) on list but without integrating scheme.
3. One cohesive statement that provides integration scheme for what follows.
4. Same as 3 but two integrating schemes.
5. Same as 4 but with clear hierarchical organization.

#### Coding creative idea expression during composing

The following coding scheme was used to assess the degree of interesting, imaginative, or insightful ideas expressed in sentences in the composition:

1. No sentences with interesting, original, imaginative, or insightful ideas expressed.
2. At least one sentence with an interesting, original, imaginative, or insightful idea expressed.
3. Two or more sentences with an interesting, original, imaginative, or insightful idea expressed.
4. Three or more sentences with an interesting, original, imaginative, or insightful idea expressed.
5. Four or more sentences with an interesting, original, imaginative, or insightful idea expressed.

#### Genres

Linguistic coding was also used to account for the kinds of genres observed in the composing outside the scanner following time to plan. The variety of genres observed is described both within the classic distinction between narrative and expository and nontraditional ones (e.g., see Kambelis, 1999, for poems as well as stories and reports). For examples of multiple genres observed denoted with case numbers please see Appendix.

#### 3.1.1. Traditional Narrative

Three kinds of narrative were observed—*first person personal narratives or stories* in which the author is the main character, *second person narrative directed to an audience of You the Reader*, and *third person narratives with fictional or historical characters* (a single person or group of persons) as is more typically associated with narrative—a sequence of events in a setting. See Case 23 for example. Sometimes the personal narrative had co-authors who are the main characters who each tell their personal stories. S*econd person narrative* in which the unfolding story is addressed to you the audience is rarer but does occur, but not as often as *third person narrative* with characters, setting, plot, and outcome about persons other than the author. Note that one participant used the idiom I think before each statement written otherwise from a third person perspective.

#### 3.1.2. Traditional Expository

Three kinds of expository genres were observed—*knowledge telling*, *knowledge transforming*, and *sharing opinions or hypotheses*. The first two are well described in the writing literature (Bereiter & Scardamalia, 1987). In knowledge telling, information or “facts” about a topic are simply listed. In contrast, in *knowledge transforming* a topic sentence makes a claim and then a case is made by citing information or other kinds of ideas selectively to support the claim rather than merely listing it. See Case 19 for example of sharing opinions or hypotheses, which is the developmental precursor to argumentative or persuasive writing first an author needs to be able to express one’s own point of view or perspective.

#### 3.1.3. Nontraditional Narrative Genres

Four kinds of narrative were observed that are not typically taught explicitly or even identified in the research literature.

##### Adventure

A story genre primarily focused around the actions of people, or actions taken against them, creating a suspenseful atmosphere because of danger or tension. See Cases 15 and 23.

##### Personal/Interpersonal Drama

A story genre where the primary tension is caused by the interpersonal relationships among people as they interact in contrast to action-oriented situations as in Adventure. See Cases 37 and 38.

##### Epistolary

A story genre focused on the internal life and thoughts of the author as a character, in which the writing reads as if it were written by that character. Typically, there is only one author character but sometimes two authors may be the characters telling their story. See Case 30.

##### Origin

A story genre that focuses on where a character came from or how the character became the person he or she is. Generally, the story is about how someone became a hero or got into an adventure. See Cases 22 and 27.

#### 3.1.4. Nontraditional Expository

Four kinds of nontraditional expository were observed. Note that although students often keep personal journals at school, there is also an expository form of journal writing in which it is designed to inform or share information with others.

Catalog this expository genre employed knowledge telling to list all the things the writer knows or can bring to mind about a topic or domain of knowledge. See Case 2.

Journal or Diary An expository genre in which the author uses dated entries in a journal or diary to describe events, share reflections, and/or provide perspectives. See Case 2.

History—An expository genre in which facts are organized along the lines of what is non-fiction in that the events described are documentable in the history of humankind. See Case 24.

Opinion or Hypothesis An expository genre in which the focus is on the opinion of the author/s or a proposed hypothesis rather than documented facts or perspectives shared by all. See Case 19 and Case 7. Both sides of an argument are not presented as in persuasive or argumentative essays.

### 3.2. Research Aim 2: Relating Coded Transcription and Translation Variables to Diagnostic Groups

#### Comparing diagnostic groups on coded transcription variables in compositions

In the process of developing the coding scheme it became evident that one participant with dysgraphia had so many illegible letters in the written composition it was not possible to code the composition. Thus, for the data analyses the dysgraphia group had an n of 13. See Table 1 for the means and standard deviations for coded handwriting quality and spelling errors for the control group, dysgraphia group, and dyslexia group. The coded transcription variables in the compositions are analogues of the normed measures used to assign participants to diagnostic groups. As also shown in Table 1, an ANOVA with diagnostic groups as a between participant variable showed a significant main effect for group for both transcription variables, handwriting quality, *F*(2, 35) = 3.23, *p* = .051, and spelling errors, *F* (2, 35) = 7.29, *p* = .002. As the follow-up *t*-tests showed, the dysgraphia group differed significantly from the control group and the dyslexia group differed significantly from the control group on handwriting quality, but the dysgraphia and dyslexia groups did not differ significantly from each other on handwriting quality. However, the dysgraphia group differed significantly from the control group and the dyslexia group differed significantly from the control group in spelling errors, but the dysgraphia and dyslexia groups also differed significantly from each other on spelling errors. The dyslexia group made over twice the number of spelling errors as the dysgraphia group. See Table 1. Thus, the first hypothesis was confirmed for transcription—handwriting quality and spelling errors.

#### Comparing diagnostic groups on translation variables in compositions

See Table 2 for the means and standard deviations of each of the observed coded variables that were coded along a quantitative scale and are organized by diagnostic groups and total sample: number of words, staying on topic, text organization, and creative expression. One-way ANOVAs with diagnostic group as a between participant variable were conducted for number of words, staying on topic, text organization, and creative expression. None of these were statistically significant, showing that an SLD related to transcription (handwriting and/or spelling) does not necessarily impair indicators of translation, although the poor quality of transcription skills may result in others underestimating the ability of a developing writer to express ideas in written language; and developing writers with transcription impairments may underestimate their own abilities in written expression of ideas apart from handwriting and spelling. Thus, the first hypothesis was not confirmed for the coded translation variables.

#### Correlations among the coded variables

Finally, correlations were computed among all six coded compositional variables. Handwriting quality was significantly correlated with number of spelling errors, *r* = −.39, *p* < .05, staying on topic, *r* = .25, *p* < .05, and text organization, *r* = .48, *p* < .01. The first was negative indicating better handwriting quality is associated with fewer spelling errors, but the other two were positive indicating that better handwriting is associated with staying on topic and better text organization. Number of spelling errors was also correlated with creative expression, *r* = .33, *p* < .01, which was curiously positive—more spelling errors associated with higher creative expression, and with higher number of words, *r* = .40, *p* < .05, which was also positive. Perhaps when focused on expressing ideas or finding words to express ideas (Stahl & Nagy, 2005), fewer cognitive resources are devoted to spelling; or the more words written, the more opportunity there is to misspell them. Staying on-topic was positively correlated with text organization, *r* = .34, *p* < .05. All these correlations were of modest magnitude, however. Only number of words and text organization, *r* = .72, *p* < .01, and creative expression and text organization, *r* = .73, *p* < .01 were of more sizable magnitude. However, none of these coded outcomes shared more than 50% of the variance in each other; so collectively they are all most likely needed to capture the multiple dimensions of the composing process.

### 3.3. Research Aim 3: Correlation between fMRI Connectivity Data and Coded Compositions

Correlations were computed for the fMRI connectivity from each of the four seed points with the targeted associated eight brain regions for a specific writing task in the scanner (see procedures—imaging) *and* specific coded variables in the compositions written outside the scanner (see Table 2). Results for correlations of statistically significant magnitude (strength) were analyzed separately for each diagnostic group. Results for transcription variables (handwriting and spelling) are reported in Table 3. Results for translation variables (number of words, on-topic, organization, and sentence creativity) are reported in Table 4. Both statistically significant and non-significant correlations are reported in Table 3 and Table 4; if significant, the number of asterisks indicates the level at which *p* is significant; no asterisk indicates non-significant.

#### Control group

The two transcription skills and planning during scanning were correlated significantly with at least one coded composition variable after scanning, but resting during scanning was not. Thus, in the control group the second hypothesis was confirmed for three writing tasks—handwriting, spelling, and planning. The statistically significant correlations for specific seed points with specific brain regions and specific coded composition variables were as follows for the control group without transcription disabilities.

*fMRI alphabet task* was significantly correlated with coded *handwriting quality* from left occipital temporal with Broca’s area, with thalamus, and with amygdala; from left supramarginal with thalamus and with amygdala; from left precuneus with Broca’s area, with left fusiform 2 and with amygdala; and from left occipital temporal with Broca’s area, with fusiform, with fusiform 2, and with amygdala.

*fMRI connectivity spelling task* was significantly correlated with coded *spelling errors* from left supramarginal with fusiform 2 and with thalamus; from left precuneus with Broca’s area, with fusiform, with fusiform 2, and with amygdala; and from left inferior frontal with fusiform.

*fMRI connectivity planning task* was significantly correlated with (a) *coded number of words* (length) from left inferior frontal with fusiform; (b) coded *on-topic* from left supramarginal with thalamus; and from left precuneus with fusiform and with cingulate; (c) coded *text organization* from left occipital temporal with fusiform 2; from left precuneus with fusiform 2, and with amygdala; and from left inferior frontal with fusiform; and (d) coded *creative expression* from left supramarginal with fusiform; and from left precuneus with hippocampus.

#### Dysgraphia group

fMRI connectivity for spelling, resting, and planning during scanning was significantly correlated with one or more coded outcomes in compositions written outside the scanner. Thus, for the dysgraphia group, the second hypothesis was confirmed for all tasks except handwriting, the impaired skill in handwriting. These correlations for specific seed points with specific brain regions and specific coded composition variables were as follows for the dysgraphia group.

*fMRI connectivity during alphabet writing and coded handwriting quality* were not significantly correlated.

*fMRI connectivity during word-specific spelling* and *coded spelling errors* were significantly correlated from left precuneus with fusiform and with amygdala; and from left inferior frontal with Broca’s and fusiform.

*fMRI connectivity during resting state* (mind wandering) was significantly correlated with (a) *coded staying on-topic* from left occipital temporal with hippocampus; from left supramarginal with hippocampus; from left precuneus with hippocampus; and from left inferior frontal with hippocampus; and (b) *coded text organization* from left precuneus with fusiform.

*fMRI connectivity during planning* was significantly correlated with (a) *coded on-topic* from left precuneus with cingulate; and from left inferior frontal with hippocampus; and (b) *coded creative expression* from left occipital temporal with Broca’s area.

#### Dyslexia group

fMRI connectivity for resting, alphabet writing, and spelling during scanning was significantly correlated with one or more coded outcomes in compositions written outside the scanner. Thus, the second hypothesis was confirmed for all writing tasks except planning in the dyslexia group. These correlations for specific seed points with specific brain regions and specific coded composition variables were as follows for the dyslexia group.

*fMRI connectivity during alphabet task* was correlated with coded *handwriting quality* from left inferior occipital temporal with thalamus; and from left supramarginal with thalamus.

*fMRI connectivity during word-specific spelling* was correlated with *spelling errors* from left supramarginal with supramarginal; and from left precuneus with supramarginal.

*fMRI connectivity during resting* was correlated with (a) coded *staying ontopic* from left inferior occipital temporal with fusiform 2; from left supramarginal with hippocampus; from left precuneus with hippocampus; and from Broca’s area with fusiform 2; (b) coded *text organization* from left precuneus with supramarginal; and (c) coded *creative expression* from left inferior occipital temporal with hippocampus; from left supramarginal with supramarginal, and with hippocampus; from left precuneus with supramarginal; and from left inferior frontal with supramarginal.

#### 3.3.1. Comparison of Patterns of Connectivity

In contrast to paradigms that focus on comparing mean differences in specific brain locations, connectivity paradigms afford an opportunity to examine patterns of interconnectedness across tasks and/or groups. Patterns of correlations on each task from each seed point were compared among the three diagnostic groups. Both the magnitude and sign of the correlations are relevant to those patterns. For the most part a positive sign indicates that the stronger the connectivity, the higher the performance on the coded composition variable. However, for spelling errors, a higher score is not a better performance and negative correlations are an indicator of better performance. Clearly, examination of patterns of significant connections in Table 3 for the different seed points with brain regions for each of the four tasks during scanning and each of two coded transcription variables or four coded translation variables after scanning show that the three diagnostic groups differ. Patterns, which are discussed separately for transcription and translation skills, show that the three diagnostic groups exhibit different profiles of internal brain connectivity on the same tasks or conditions.

##### Transcription

For ***handwriting***, the **control group** showed significant correlations from each seed point (three each for three seed points and two for the fourth seed point); the **dysgraphic group** showed none; and the **dyslexic group** showed significant correlations from two seed points (one from each seed but only one of which was the same as for the control group—from left supramarginal with thalamus). For ***spelling***, the **control group** showed significant correlations from three seed points (all but left occipital temporal) which were connected with two, four, or one other brain region; the **dysgraphia group** showed significant correlations with three of the same (left precuneus and fusiform; left inferior frontal and Broca’s area and fusiform)—but only these three; the **dyslexia group** showed only two significant correlations not shared with either the control or dyslexia group—left supramarginal and left precuneus with supramarginal. See Table 3.

##### Translation

For ***resting* (*mind wandering*) *and number of words***, none of the groups showed a significant correlation. For ***mind wandering and staying on-topic***, the **control group** showed no significant correlations; but the **dysgraphia group** was significantly correlated with hippocampus (associated with working memory) from each of the four seed points; and the **dyslexia group** was significantly correlated with fusiform 2 (associated with written words) from the left occipital temporal seed and with hippocampus (associated with working memory) from the left inferior frontal seed. For ***mind wandering and text organization***, the **control group** showed no significant correlations; the **dysgraphia group** showed a significant correlation only for connectivity from left precuneus with fusiform; and the **dyslexia group** showed a significant correlation only for connectivity from left precuneus with supramarginal. For ***mind wandering and creativity***, neither the **control group** nor the **dysgraphia group** showed significant correlations, but the **dyslexia group** showed significant correlations from the left occipital temporal and from left supramarginal with hippocampus, from all but the left occipital temporal with supramarginal, and from left inferior frontal with fusiform 2. Thus with the exception of the control group and dysgraphia group for creative expression, the three diagnostic groups did not show the same pattern of correlations for resting during scanning and coded composition variables after scanning. See Table 3.

For ***planning* (*strategic prior to composing*) *and number of words***, only the **control group** showed a significant correlation with composing after scanning. For ***planning and staying on-topic***, the **control group** showed significant correlations with connectivity from left supramarginal with thalamus, and from left precuneus with fusiform with cingulate; the **dysgraphia group** showed significant correlations from left precuneus with cingulate, as had the control group, and from left inferior frontal with hippocampus, which the control group did not; and the **dyslexia group** showed significant correlations from left occipital temporal and left supramarginal with cingulate, a completely different pattern from the other diagnostic groups. For ***planning and text organization***, only the **control group** showed significant correlations; these were from left occipital temporal and from left precuneus with fusiform 2 and from left precuneus with amygdala. For ***planning and creativity***, the **control group** showed significant correlations from left supramarginal with fusiform and from left precuneus with hippocampus; the **dysgraphia group** showed significant correlation from left occipital temporal with Broca’s; and the **dyslexia group** showed no significant correlations.

Thus, the diagnostic groups generally showed different patterns of correlations. The only exceptions were that both the dysgraphia and dyslexia groups differed from the control group on planning and number of words and planning and text organization; and the dysgraphia and control groups shared one common significant correlation. See Table 3.

## 4. Discussion

### 4.1. Implications for Creative Expression in Written Language

Findings for coding the genres observed in the written compositions outside the scanner are consistent with other recent research showing diversity in written compositions beyond the traditional narrative and expository distinction (Boscolo, Gelati, & Galvan, 2012; Davidson & Berninger, 2015; Moore & McArthur, 2012; Olinghouse, Santangelo, & Wilson, 2012; Olinghouse & Wilson, 2013). The coding divided nontraditional narrative into adventure, epistolary, origin, and personal drama, and nontraditional expository into Catalog, Opinion or Hypothesis, History, Journal or Diary. First person, second person, and third person narratives.

Careful examination of the variety of cognitions expressed and genres for doing so illustrates the diversity. Several narrative genres observed closely tracked with those in popular film, tv, and literature. Students’ narratives about astronauts sometimes took the form of adventure stories, where the astronauts took action, and suspense was created through physical danger. These stories included running out of gas, lasers, and guns. One student wrote “Not waiting, they run out the door, though going one at a time: they climb down the ladder, and into the rocky biome, feeling a little light headed… None the less they continue on, through the barren landscape.” See full case 25 in the Appendix, and another adventure in which Neptune is entirely overrun by giant sharks (Case 23).

A Personal Drama, unlike action, focuses the primary reason for conflict on interpersonal interactions, rather than actions, shootings, or physical stressors. These interpersonal interactions may include wonder “They knew that they would never be able the forget this moment,” (Case 38) or personal accomplishment “It was a long difficult rode for Jan. She had to go through alot more training then Steve did.” (Case 37). See Appendix.

An Origin narrative focuses on where a character came from or how they became the person they are. Generally, the story is about how someone became a hero or got into an adventure. These are very similar to the currently popular movies that tell how a person came to be a hero. For example, case 27 ends with the end of training to determine “if you can withstand 6 months in a shuttle.” See cases 22 and 27 in the Appendix.

Of particular interest were a few epistolary compositions which told the narrative of an astronaut, as if the astronaut was the one writing the text itself. These stories included many subtle cues that integrated the themes of astronauts and writing. For example, we noticed that these “journals” were often dated with fictitious dates, and included commentary on the purpose of the journal. In just one example, in case 2, the writer says “It was recommended that we all write journals of our travels and, seeing as I didn’t want to disappoint, I followed the advice. So this is my journal of my journey in space.” Here, the author portrays the writer with a motivation and an audience. See cases 2 and 30 in the Appendix.

Expository compositions also gave evidence of a variety of methods for organization. The simplest of these, which we coded as a “catalog” included significant “knowledge telling”—in which students simply repeated all the knowledge they had about astronauts with no particular structure or order. A representative passage is “astronauts eat food and these are some of them space ice cream and alot of dried foods they cant eat alot of normal foods that we eat because it would evaparate right away Some fun things you could do in space ship are you could float around with no gravitie and maybe get a basketball hoop in the space ship” from case 1, but also see others included in cases 6 and 9 in the Appendix. Other compositions focused primarily upon the perspective of the author—these we termed opinion, and are illustrated by cases 19 and 7. One composition we coded as history, Case 24, which occurred less frequently, but points to the inherent generativity of genre at the discourse level. Collectively, the results show that students employed many more genres than typically taught at school (e.g., Cutler & Graham, 2008).

On the one hand, this genre diversity may stem, in part, from the composing process, like the writing brain itself, undergoing changes across development and schooling (Epstein-Jannai, 2004; Gillespie, Olinghouse, & Graham, 2013; Olive, Favart, Beauvais, & Beauvais, 2009). For example, in the current study of developing writers in grades 4 to 9 there were no in-depth examples of compositions reflecting a well-formed argument integrating knowledge into a pro and con argument or attempt to convince, possibly because that is a skill that may not be mastered until later in high school or even postsecondary years. On the other hand, this genre diversity may stem from the generativity of language not only at the syntax level (Chomsky, 2006) but also at the genre level of cognitive-translation. That is, genre is a word that captures the generativity of language at the text or discourse level such that written composition products at this level of language are not easily categorized simply as narrative or expository. Moreover, the rich and varied expression of thought in written language at the text level serves as a reminder that clinical and psychoeducational assessment benefits from not only including normed measures but also going beyond a single normed measure of written composing to analyzing the kinds and quality of translation indicators in compositions students produce.

### 4.2. Implications for Understanding Transcription Disabilities in Dysgraphia and Dyslexia (Research Aim 2)

Whereas some children met the research criteria for impaired handwriting (dysgraphia) others met the research criteria for impaired word spelling (dyslexia)—on normed measures, and the test results were consistent with parent reports of current and past history of these specific kinds of transcription disabilities. When groups with transcription disabilities were compared on coded transcription variables in their written compositions outside the scanner, both groups differed from the control group on coded handwriting quality and spelling errors; however, the dysgraphia and dyslexia group differed from each other only on spelling errors, with the dyslexia group making nearly twice as many spelling errors as the dysgraphia group. This finding is consistent with dyslexia being a spelling disability and not just a reading disability (Bruck, 1993; Connelly, Campbell, MacLean, & Barnes, 2006; Lefly & Pennington, 1991). This pattern of results is also consistent with the cascading levels of language diagnostic framework (Berninger et al., 2015). Whereas both those with dyslexia and dysgraphia may be impaired in lower level subword letter writing, only those with dyslexia have primary impairment in the higher level word spelling and reading (Berninger et al., 2015). These coded composition results for transcription variables support the construct validity of using both normed measures and careful examination of handwriting and spelling in composition samples in diagnosing dysgraphia and dyslexia.

### 4.3. Implications for Trends in Neuroscience for Brain-Behavior Relationships in Writing (Research Aim 3)

Much brain research on typically developing language learners and those with SLDs in written language learning has focused on single regions of interest (ROI’s) associated with particular functions. However, the recent paradigm shift to the human connectome (Smith, 2012; Sporns, 2013; Richards et al., 2015) is showing that brain functions are more complex than a single region being associated with a single function or a single network of connectivity being involved in a function. Rather, regions in different places in the brain may co-activate at the same time that an ROI, referred to as a seed point, does, and more than one network of such connectivity may support a given task or condition. Connectivity paradigms can supplement, not replace, what has been learned from R01 alone studies. On the one hand, correlations between brain connectivity and writing products may inform a rapidly expanding body of research showing that on-line processing prior to written production plays an important role in written composing (Alamargot, Chesnet, Dansac, & Ros, 2006; Alves & Limpo, 2015; Chenoweth, & Hayes, 2003; Leijten, & Van Waes, 2013). On the other hand, these correlations of brain connectivity and writing production may inform understanding of the underlying processing differences between children and youth with different kinds of transcription disabilities. In the current study the three diagnostic groups studied—typical controls, dysgraphia, and dyslexia—differed in patterns of connectivity in the scanner depending on the brain imaging task they were performing for transcription or translation and they differed in coded transcription or translation variables outside the scanner.

#### Significance of findings for control group

The significant correlations between connectivity from three seed regions associated with written language (spelling and reading words) or precuneus in the default network during scanning for alphabet writing task and handwriting during composing shows that handwriting is not just a motor skill—written language at the subword level and related processes are involved. Some of these correlations involved connectivity with regions associated with language (Broca’s) and letters in written words (fusiform and fusiform 2). The correlations for connectivity with thalamus on the handwriting task show attention also may play a role in handwriting. Of interest, correlations were observed between all four seed points on the alphabet task during scanning with amygdala and the coded handwriting quality after scanning; this brain region is associated with emotional response, suggesting that for even those without a handwriting disability producing quality handwriting may be associated with an emotional response. However, future research might investigate whether in the case of those without transcription disabilities the amgydala connection is related to approach in the approach-avoidance gradient but in those with transcription disabilities to fear and avoidance.

Spelling errors in compositions were correlated with connectivity from all seed points, except left occipital temporal, with fusiform, which has been well documented in brain imaging studies to be involved in written word learning (see Cohen, Lehéricy, Chochon, Lemer, Rivaud, & Dehaene, 2002; James, Jao, & Berninger, 2015; Longcamp, Richards, Velay, & Berninger, 2017). Of importance, for the two cognitive conditions, none of the coded compositions variables was correlated with resting state connectivity (mind-wandering) in the control group. Connectivity with cingulate, known to be involved in self-regulation and executive functions, was observed only in the correlation between connectivity for planning during scanning and staying on-topic in composing after scanning.

#### Significance of findings for dysgraphia group

Of note, considering the hallmark impairment in dysgraphia is writing alphabet letters, none of the correlations for alphabet writing during scanning was significantly correlated with handwriting quality after scanning. Behavior outside the scanner was dissociated from processing in the scanner. Two of the correlations for spelling involved fusiform, which is known to activate during spelling tasks. Correlation was also found between connectivity with cingulate, which is associated with executive functions, and coded staying on-topic during composition.

#### Significance of findings for dyslexia group

For transcription skills—handwriting and spelling, correlations were found between connectivity during scanning and coded variables after scanning. However, no correlations were found for planning during scanning and coded composing variables after scanning. Coded translation variables correlated only with resting condition (mind wandering, Raichle et al., 2001). This pattern of findings suggests that during composing writers with dyslexia may be in flow (Kellogg, 1994) rather than engaging in strategic planning (Hayes, 1996; Shah et al., 2103).

### 4.4. Implications for Educational Neuropsychology Assessment and Writing Instruction

The goal of clinical assessment is often to develop instructional plans. Much has been learned about effective, evidence-based instructional approaches for teaching struggling writers (MacArthur, Graham, & Fitzgerald, 2015; Troia, 2009). The current research suggests that instruction might be tailored to support the diversity of genres observed in the coded written compositions in the current study. Strategies used to teach struggling writers to self-regulate their composing have often focused primarily on narrative versus expository. For example, Gilbert and Graham (2010) differentiated “storytelling” as narrative, and journaling, but did not address their combination, or the varieties within each of them. Gillespie, Olinghouse, and Graham (2013) found that students’ knowledge of genres correlated with knowledge about substantive writing processes, but this understanding of genres varied strongly. If these genres are generative, then teachers can benefit from understanding their differences and guiding students to develop self-regulation strategies to write in a variety of genres and encourage creativity of expression beyond conventional genres.

Moreover, students with dysgraphia and dyslexia may need explicit instruction in transcription embedded in their learning activities for self-regulating translation during composing. For those with dysgraphia, the transcription instruction should focus on self-regulating their handwriting For those with dyslexia, who are more likely to engage in flow than pre-planning, the transcription instruction should focus on both spelling and handwriting, and also strategic planning (self-regulated composing, Harris, Graham, Mason, & Friedlander, 2008). In conclusion, behavioral cognitive and linguistic analyses of composition products, when coupled with brain research, can inform instructional practices.

### 4.5. Limitations, Future Directions, and Conclusions

Considering the numerous potential connections from various seed points in the brain for a given task, the risk of false positives and false negatives exists. Only as connectivity paradigms, which analyze *patterns of multiple connections*, are applied to other samples with well-defined inclusion criteria, analyzed at both the group and individual levels, and findings replicate, can firm conclusions be reached about correlations between brain connectivity of developing children and youth with and without transcription disabilities and their written productions. At the same time it is important that future research investigate the potential patterns in connections in neural networks in the highly connected, complex human brain or otherwise risk the error of oversimplifying and assuming single locations in the brain or single processes explain writing or that brain-behavior relationships are same in writing for all children and youth. The current study is a beginning step that shows the promise of integrating brain research to assess on-line, internal processing with behavioral research that codes cognitive and linguistic variables in external written output.

In conclusion, however, the observed complexity and rich diversity of the translation processes in the coded compositions following planning in the brain scanner support the process approach to clinical psychoeducational assessment that includes but does not rely exclusively on normed measures. Evidence-based assessment considers multiple writing samples and multiple aspects of diverse genres and other indicators of translation, both in individuals with and without transcription disabilities.

## Figures and Tables

**Table 1:** Behavioral assessment based on coded transcription skills in compositions written after scanning for each diagnostic group.

	Control Group		Dysgraphic Group		Dyslexic Group		(df) t	p

	M	SD	M	SD	M	SD		
**Handwriting quality**	3.22	0.67	2.54	0.89	2.44	0.89		
control vs. dysgraphia							(20) 2.38	.03
control vs. dyslexia							(23) 2.30	.03
dysgraphia vs. dyslexia							(27) .34	.74 ns
**Spelling errors**	2.78	2.95	7.08	4.59	16.09	13.64		
control vs. dysgraphia							(20) −2.47	.02
control vs. dyslexia							(23) −2.99	.006
dysgraphia vs dyslexia							(27) −2.43	.02

**Table 2:** Means and standard deviations for each coded category on a quantitative scale in compositions following scanning, organized by diagnostic groups.

Diagnostic Group	Coded Category	M	SD
**Control (N = 9)**	Handwriting Quality	3.22	0.66
	Number Spelling Errors	2.78	2.95
	Number of Words	155.1	171.68
	Staying On-Topic	4.00	1.00
	Text Organization	3.00	1.58
	Creative Expression	2.67	1.32
**Dysgraphia (N = 13)**	Handwriting Quality	2.54	0.66
	Number Spelling Errors	7.08	4.59
	Number of Words	127.31	106.81
	Staying On-Topic	3.46	0.78
	Text Organization	2.46	1.13
	Creative Expression	2.62	1.45
**Dyslexia (N = 16)**	Handwriting Quality	2.44	0.89
	Number Spelling Errors	16.69	13.64
	Number of Words	140.31	93.35
	Staying On-Topic	3.31	0.87
	Text Organization	2.75	1.39
	Creative Expression	2.75	1.48
**Total (N = 38)**	Handwriting Quality	2.66	0.82
	Number Spelling Errors	10.11	10.91
	Number of Words	139.37	91.96
	Staying On-Topic	3.53	0.89
	Text Organization	2.71	1.33
	Creative Expression	2.68	1.40

**Table 3:** Correlations between functional connectivity from 4 seeds (rows) with 8 brain regions (columns) on 2 writing tasks during scanning and 2 coded transcription variables in composing outside the scanner (see table subheadings) by 3 diagnostic groups.

	3.1. ALPHABET IN AND OUT OF SCANNER							

**GROUP**	**with Broca’s**	**with fusiform**	**with supramarginal**	**with fusiform 2**	**with cingulate**	**with thalamus**	**with hippocampus**	**with amygdala**

**CONTROL**								
**LOCT**	−.66*	.28	−.47	−..20	−.06	.67 *	−.22	.83**
**LSupra**	−.35	.07	.35	−.43	.54	.68*	−.30	.67*
**LPre**	−.81**	−.64	−.14	−.71*	−.36	.61	−.63	.76*
**LIFG**	.81**	−.72*	.48	−.71*	−.43	.10	.03	.89***
**DYSGRAPHIA**								
**LOCT**	−.04	−.43	−.11	−.37	.01	.11	−.25	.21
**LSupra**	.26	−.43	.01	.07	−.17	.34	−.25	.21
**LPre**	−.32	−.18	−.44	−.28	.06	−.49	−.25	.09
**LIFG**	−.08	−.23	−.35	−.08	.07	.02	−.50	.28
**DYSLEXIA**								
**LOCT**	−.50	.42	−.01	−.03	−.20	−.60*	.12	−.11
**LSupra**	−.03	−.16	−.04	−.46	−.39	−.63**	−.17	−.12
**LPre**	−.29	−.36	.10	.46	−.14	−.31	.01	.07
**LIFG**	−.02	−.20	−.150	−.43	−.42	−.00	−.31	−.04

	3.2. SPELLING IN AND OUT OF SCANNER							

**GROUP**	**with Broca’s**	**with fusiform**	**with supramarginal**	**with fusiform2**	**with cingulate**	**with thalamus**	**with hippocampus**	**with amygdala**

**CONTROL**								
**LOCT**	−.04	−.10	.29	.54	−.26	−.37	−.34	.34
**LSupra**	.17	.49	−.17	.84**	−.57	−.68*	−.60	−.29
**LPre**	.73*	.74*	.33	.94***	.15	−.34	−.35	−.94***
**LIFG**	−.08	−.85**	.55	−.28	−.06	−.60	−..60	−.41
**DYSGRAPHIA**								
**LOCT**	−.18	−.06	.30	−.20	−.05	.19	.54	−.18
**LSupra**	.04	−.24	.51	.12	.45	−.01	−.24	−.20
**LPre**	.11	−.61*	−.08	−.06	.29	−.27	.01	−.54
**LIFG**	−.69**	−.60*	.03	−.20	−.00	−.06	−.49	−.26
**DYSLEXIA**								
**LOCT**	.17	.04	−.38	−.19	−.22	−.07	−.46	−.00
**LSupra**	.13	−06	−.69**	−.01	−.01	−.25	−.05	.21
**LPre**	.18	.15	−.52*	−.16	−.35	−.19	−.28	.06
**LIFG**	−.23	−.14	−.36	.08	.05	−.362	−.15	−.12

Notes. Seeds: LOCT = left occipital temporal; LSupra = left supramarginal, LPre = left precuneus, LIFG = left inferior frontal gyrus (Broca’s). Tasks during Scanning: Alphabet Writing, Spelling (See procedures—imaging) Coded Compositional Variables: Handwriting Quality, Spelling (See Table 2).

* p < .05,

** p < .01,

*** p < .001, ns = p > .05. See table notes.

**Table 4:** Correlations between functional connectivity from 4 seeds (rows) with 8 brain regions (columns) on 2 writing tasks during scanning and 4 coded translation variables in composing outside the scanner (see table subheadings) by 3 diagnostic groups.

	4.1. (a) MIND WANDER AND WORDS							

**GROUP**	**with Broca’s**	**with fusiform**	**with supramarginal**	**with fusiform 2**	**with cingulate**	**with thalamus**	**with hippocampus**	**with amygdala**

**CONTROL**								
**LOCT**	−.05	−.31	.28	.32	.42	.41	.45	.38
**LSupra**	.15	.13	.33	.33	.36	−.11	.25	.11
**LPre**	.32	−.28	.181ns	−.24	.43	.10	.35	.23
**LIFG**	.32	.40	.04	.01	.52	.06	.35	.10
**DYSGRAPHIA**								
**LOCT**	.02	.03	.06	.11	.24	.30	.51	−.16
**LSupra**	.14	.08	−.07	.19	.12	.15	.31	.01
**LPre**	.31	−.28	−.01	.11	−.07	−.04	.13	−.08
**LIFG**	.08	.02	−.16	.12	.26	.23	.13	−.14
**DYSLEXIA**								
**LOCT**	.45	−.03	−.26	.13	.24	−.43	−.27	.17
**LSupra**	.45	.03	−.40	.38	.36	−.48	−.23	.18
**LPre**	−.04	.18	−.43	−.23	.01	−.43	−.23	−.05
**LIFG**	.24	.12	−.45	.23	−.04	−.46	−.06	.18

	4.2. (a) MIND WANDER AND ON-TOPIC							

**GROUP**	**with Broca’s**	**with fusiform**	**with supramarginal**	**with fusiform 2**	**with cingulate**	**with thalamus**	**with hippocampus**	**with amygdala**

**CONTROL**								
**LOCT**	−.36	.22	−.14	.38	.21	.01	−.45	−.17
**LSupra**	−.34	−.06	−.16	.21	.10	.18	.09	−.10
**LPre**	.04	.20	.38	.44	.20	.14	−.61	.06
**LIFG**	.32	.40	.04	.01	.52	.06	.35	.10
**DYSGRAPHIA**								
**LOCT**	−.08	−.06	−.15	.41	.05	.02	.63*	−.38
**LSupra**	.21	.12	−.20	.17	−.34	.06	.67**	−.11
**LPre**	.03	.06	−.13	−.05	−.29	−.15	.57*	−.19
**LIFG**	.22	.12	−.38	−.05	−.29	−.15	.57*	−.19
**DYSLEXIA**								
**LOCT**	.07	.42	−.24	.59*	.25	−.04	−.48	.19
**LSupra**	−.17	.26	−.11	.47	.49	−.18	−.63**	.32
**LPre**	−.19	.16	−.47	.13	−.10	−.21	−.54*	−.07
**LIFG**	−.14	.31	−.21	.63**	−.19	−.09	−.47	−.40

	4.3. (a) MIND WANDER AND ORGANIZE							

**GROUP**	**with Broca’s**	**with fusiform**	**with supramarginal**	**with fusiform 2**	**with cingulate**	**with thalamus**	**with hippocampus**	**with amygdala**

**CONTROL**								
**LOCT**	.05	−.39	.40	.23	.38	.44	.36	.46
**LSupra**	.03	.04	.45	.36	.39	.17	.13	.21
**LPre**	.32	−.51	.09	−.31	.49	−.03	.25	.32
**LIFG**	.17	.20	.05	−.04	.50	.00	.02	.18
**DYSGRAPHA**								
**LOCT**	.17	−.10	.14	.04	.17	.41	.18	−.03
**LSupra**	.09	−.12	.10	.10	.00	.15	.05	.09
**LPre**	.36	−.59*	.10	.01	−.03	−.12	−.07	.17
**LIFG**	.23	−.01	.10	.20	.48	.22	−.03	−.08
**DYSLEXIA**								
**LOCT**	.13	.29	−.35	.44	.28	−.30	−.25	.11
**LSupra**	.13	.07	−.41	.28	.45	−.43	−.41	.15
**LPre**	−.30	.26	−.57*	−.21	.02	−.41	−.21	−.12
**LIFG**	−.05	.17	−.41	.42	−.19	−.38	−.23	.25

	4.4. (a) MIND WANDER AND CREATIVITY							

**GROUP**	**with Broca’s**	**with fusiform**	**with supramarginal**	**with fusiform 2**	**with cingulate**	**with thalamus**	**with hippocampus**	**with amygdala**

**CONTROL**								
**LOCT**	−.10	−.33	.15	−.08	.35	−.30	.22	.48
**LSupra**	−.07	−.06	.14	−.02	.32	−.19	−.05	.23
**LPre**	.10	−.32	−.27	−.50	.43	−.08	.20	.28
**LIFG**	.08	.05	−.10	−.43	.51	.09	.03	.28
**DYSGRAPHIA**								
**LOCT**	−.01	−.20	−.03	−.11	−.04	.44	.09	.23
**LSupra**	−.23	−.24	.10	.13	−.13	.11	.01	.27
**LPre**	.41	−.46	.12	.23	.26	.04	.04	.30
**LIFG**	−.03	−.16	.26	.18	.22	.33	−.11	−.03
**DYSLEXIA**								
**LOCT**	.23	.12	−.39	.37	.16	−.24	−.54*	.18
**LSupra**	.13	−.01	−.53*	.43	.31	−.37	−.55*	.14
**LPre**	−.21	.09	−.66**	.07	−.36	−.31	−.44	−.08
**LIFG**	.04	.23	−.63**	.56*	−.25	−.26	−.26	.36

	4.1. (b) PLANNING AND WORDS							

**GROUP**	**with Broca’s**	**with fusiform**	**with supra-marginal**	**with fusiform2**	**with cingulate**	**with thalamus**	**with hippocampus**	**with amygdala**

**CONTROL**								
**LOCT**	−.51	.60	.25	−.55	.20	.16	.00	. 13
**LSupra**	−.35	.44	−.18	.46	−.17	−.39	.41	.04
**LPre**	−.48	.01	.13	−.52	.18	−.09	−.25	.47
**LIFG**	−.08	−.68*	.06	−.04	−.09	.18	.25	.26
**DYSGRAPHIA**								
**LOCT**	.40	.00	.32	.17	.34	.08	.41	−.02
**LSupra**	.10	.44	−.04	.26	.04	.30	−.14	.15
**LPre**	.37	−.13	.24	−.04	−.19	−.00	.46	−.19
**IFG**	.19	.20	.12	.32	.45	−.03	.30	−.15
**DYSLEXIA**								
**LOCT**	.17	−.05	−.19	−.13	−.05	−.46	−.12	−.01
**LSupra**	.15	−.10	−.45	−.09	.50	−.28	.02	.05
**LPre**	−.02	.04	−.45	−.01	−.31	−.48	.01	−.32
**LIFG**	.08	−.40	−.10	.00	.28	−.37	−.00	.18

	4.2. (b) PLANNING AND ON TOPIC							

**GROUP**	**with Broca’s**	**with fusiform**	**with supramarginal**	**with fusiform 2**	**with cingulate**	**with thalamus**	**with hippocampus**	**with amygdala**

**CONTROL**								
**LOCT**	.26	.14	−.10	−.05	.63	.47	.38	.21
**LSupra**	.34	−.51	.05	.20	.60	.67*	.32	−.48
**LPre**	.11	.94***	.04	−.05	.71*	.46	.33	.12
**LIFG**	.02	.31	−.58	.39	.52	−.04	−.00	.03
**DYSGRAPHA**								
**LOCT**	.04	.36	.23	.53	.38	−.12	.47	−.06
**LSupra**	.29	.14	.15	.09	.21	.51	.01	.08
**LPre**	.29	.01	.11	.13	.60*	−.07	.29	−.19
**LIFG**	.31	.31	.13	.24	.16	.09	.81***	.08
**DYSLEXIA**								
**LOCT**	.47	.07	.27	.18	.57*	.08	−.12	.07
**LSupra**	−.15	.18	−.01	.41	.57*	.09	−.17	.34
**LPre**	.09	.23	−.06	.45	.12	−.02	−.03	−.01
**LIFG**	−.07	−.03	.31	.27	.45	−.05	−.43	.40

	4.3. (b) PLANNING AND ORGANIZE							

**GROUP**	**with Broca’s**	**with fusiform**	**with supramarginal**	**with fusiform 2**	**with cingulate**	**with thalamus**	**with hippocampus**	**with amygdala**

**CONTROL**								
**LOCT**	.37	.41	.18	.67*	.30	.27	.08	.27
**LSupra**	−.50	.29	−.37	.35	−.15	−.56	.56	−.27
**LPre**	−.65	−.27	−.52	−.67*	.05	−.23	−.50	.67*
**LIFG**	−.37	−.66*	−.10	−.44	−.22	.24	.23	.33
**DYSGRAPHIA**								
**LOCT**	.45	−.03	.05	.15	.02	.07	.32	.20
**LSupra**	−.14	.30	−.04	.02	−.32	.09	.11	.00
**LPre**	.19	−.37	.27	−.01	−.39	−.13	.01	.12
**LIFG**	.16	−.17	−.04	.41	.03	−.12	−.11	.01
**DYSLEXIA**								
**LOCT**	.23	−.02	−.17	−.07	.25	−.20	−.10	−.11
**LSupra**	.03	.12	−.39	.11	.50	−.11	−.01	.10
**LPre**	.00	.23	−.49	.21	−.26	−.39	.01	−.32
**LIFG**	.10	−.17	.08	−.00	.40	−.12	−.03	.25

	4.4. (b) PLANNING AND CREATIVITY							

**GROUP**	**with Broca’s**	**with fusiform**	**with supramarginal**	**with fusiform 2**	**with cingulate**	**with thalamus**	**with hippocampus**	**with amygdala**

**CONTROL**								
**LOCT**	.55	.21	.08	−.33	−.08	−.03	−.25	.40
**LSupra**	−.56	.68*	−.22	.27	−.28	−.63	.57	−.07
**LPre**	−.49	.26	.45	−.48	−.29	−.43	.71*	.41
**LIFG**	.39	−.60	.14	−.32	−.28	.27	.19	.44
**DYSGRAPHIA**								
**LOCT**	.73**	.05	.35	.21	.23	.36	.37	.56
**LSupra**	.09	.53	.18	.13	.06	.07	.41	.14
**LPre**	.43	−.05	.36	.16	−.33	.25	.13	.38
**LIFG**	.26	.13	.09	.50	.23	.10	−.21	.26
**DYSLEXIA**								
**LOCT**	.16	−.21	−.24	−.16	.05	−.27	−.30	−.02
**LSupra**	.01	−.14	−.30	.08	.41	−.16	−.12	.16
**LPre**	−.06	.04	−.49	.20	−.36	−.33	−.12	−.27
**LIFG**	−.24	−.37	−.19	.07	.38	−.43	−.28	.36

Notes. Seeds: LOCT = left occipital temporal; LSupra = left supramarginal, LPre = left precuneus, LIFG = left inferior frontal gyrus (Broca’s). Tasks: during Scanning: Resting (Mind Wandering) and Planning (see procedures-imaging) Coded Compositional Variables: Words, On-Topic, Organize, Creativity (see Table 2).

* p < .05,

** p < .01,

*** p < .001, ns = p > .05. See table notes.

## Appendix: Cases (Writing Samples of Participants) Illustrating Different Genres

### Note that the spellings are those that the students used and illustrate the nature of the SLDs-WL that many had in transcription (handwriting and/or spelling)

Adventures:

Case 15:

Many groupes of people have made it to the moon, and back. Space travel is not necessarily “all fun in games,” though I have never been there myself, it’s pretty easy for me to assume that some astraunaghts have even lost their lives going into space, some have come pretty close to, Not waiting, they run out the door, though going one at a time: they climb down the ladder, and into the rocky biome, feeling a little light headed… None the less they continue on, through the barren landscape. It seems there is so much to do, so many interesting things to attend to. They take a moment to look out the tiny each, which is heard to think is actually their home… Nothing can compare to being that “high up,” or …on a whole other planet! Lot’s of assumptions in this…

Case 23:

Long into the future, humans had learned how to make Mars be able too live on. But the population was growing to big for just Earth and Mars. They needed another Planet. From 25 astronaughts they chose 3. Those 3 were to travel our universe in search for another Planet. They could only bring 1 small bag of supplies. They had exactly 2 month before mission control would have auto pilot bring the rocket back. That night they set out on the missoin. First they went to Jupiter but their were hundreds of little aliens storming over the planet. Next they tried Saturn but giants ran through. Then they tried Neptune, which was overrun by giant Sharks. It had been 2 month and they had failed the mission. =(

Personal Dramas:

Case 37:

Jan and Steve had been waiting for this day, all the training would finally pay off. Jan and Steve were on there way to outer space. This had been steves dream his whole life and he couldn’t believe he was actually going to get to do it. Jan was just as excited as Steve, even with her Autism the program letting her go in to space was amazing. It was a long difficult rode for Jan. She had to go through alot more training then Steve did. even with the hard, sometimes painful training Jan was having the time of her life. She couldn't wait until she made it into space.

Case 38:

Whoosh! Amanda and Frank zoomed through space, amazed at what they were seeing. All their years of training hadn’t prepared them for the glorious sights they were seeing, and no amount of studying would be able to prepare anyone for it. They knew that they would never be able the forget this moment, or the feeling the acceleration of the rocket gave them. They stared in awe at the stars, as they had never realised, no matter how many facts they knew, how many and how large they were. Amanda needed to document it, and she pulled out her diary and wrote: “Wow! I’ve never seen anything like this, the Stars and everything is just beautiful! I can’t believe how much I’ve lived without knowing about all of this.” Amanda. At that moment, Amanda and Frank knew that they would tell their children and grand children of this amazing journey, and everything that they experiences during it. They would never see the sky the same way again, never without remembering that exact instant in a whole universe of time.

Origin:

Case 22:

Asoatnts are pepole who are mainly evolved in the exporsyen of space. in order to become a Astornot you first must go thouextasive testing and trianing. if you luky (and smart enof) endf to get into nasa deep space exprsheu program; you have to be phicayl fit and mentaling capable. thephical part is seeing if your body can stand zero gravity and tasks that the mission reauie. the three main ways they test that is with eating dehdreted space food and dieting. to test zero gravet they use a vertical tuke of macie it spins you at the speed of a take of. for the mental capasty they loock you in a capusle the size of the shuttle for 3 weeks in order see if you can withstand 6 months in a shuttle.

Case 27:

astronat are peole that travle to space and study outer space. Jim is going to outer space hestudyed outer space for a long time. he’s going right now. 10, 9, 8, 7, 6, 5, 4, 3, 2, 1, Blast off. He gose to outer space and see millon’s of star’s every were He look’s and see erath then He See’s the moon look cold and Dark But light on won side he landed on the moon steped off and jumped there was now gravity. So He jump Supper High. Crush, Crush the growed was covered in rock’s.

Epistolary:

Case 30:

Did you know that almost 50% of the interior of a space shuttle is taken up by paper? I should know, I was in one. Hi, my name is Eddie Emmerson. I’ll bet you’ve never heard of me. Yeah, that’s because the crew I went with, back in 2025, to Mars with (a pit-stop at the moon along the way) didn’t see it fit to list me or credit me on anything. I suppose that may be because I wasn’t actually an astronaut-I was, well, pretending. By now you’re probably wondering two things (or maybe more there’s really no way to be sure): one, why was I pretending to be an astronaut, & two, why I gave that seemingly random fact at the beginning when I was aboard the “Moon Launcher VI,” I tried sending messages back to Earth & naturally the only way to do so from space is to write it on a giant piece of paper, fold it into abnormally large paper airplane, throw it into space as hard as possible. Did ground control get the letters? I don’t know. I was trying to apologize for taking the plane of their captain on board, but I don’t think they liked it because I never heard anything back! You see, pretending to be someone else is what I do …ish. We were abroad the space craft when they found out I was an impostor. Right after lift-off I apparently didn't take the proper precautions & blah, blah, blah. They demanded to see my license. “Oh, my pilot’s license? Revoked. Or maybe you’re taking about my license to kill-also revoked. I would tell you why, but then I’d have to kill you, which I cannot do since m license to kill has been revoked.”

Knowledge Telling:

Case 1:

Space astronauts who are space traveling do some of these things they write regular and record them talking and also they send videos to earth so they can communicate with people astronauts eat food and these are some of them space ice cream and alot of dried foods they cant eat alot of normal foods that we eat because it would evaparate right away Some fun things you could do in space ship are you could float around with no gravitie and maybe get a basketball hoop in the space ship and dunk with know air When you are on the Job it is hard you have to examine rocks and stones and look at this machine that takes pictures of any thing

Case 6:

To start your carrer as a as trognot you spin around in this thing that looks something like this.

(picture)

you spin in it until you dont barf. thenou get put in a space sute and you get put in a tank of water and do some tasks and if your good eghoufe you get sent into space.

Case 9:

Astornots explore spase many different ways. The most common is the astornots send rovers, in to spare instead of them, because, the astornots don’t know if it’s to dangres to go into space. so they send rovers to like check to see if it's okay to go to that place the astornotswhats to go.

Also the Astornots can go up there them selvez, but they whould have to be prepared for all situations, But they usually send rovers.

Opinion:

Case 19:

I think austronauts have one of the coolest jobs, but I wouldn’t want to be one because they have to go through years of tough preperation and I really don’t like space. I believe that astronauts have one of the hardest jobs. I think they have hard jobs because they have to be able to by around a 300 pound suit and still be able to do everythings. I think astrounats wouldn’t handwrite things but they would type things. I think that in the next 25 years we will send someone to Mars. I think that eventually scientist will fin away for many other planets to be suitable for life. I also think that in the next 25 years people are going to prove that their are aliens.

Case 7:

astronauts in space have a very dangrous job because they are in space. That is why they send messages while they are in space. they can keep in touch and know whats happening down, on earth and ask for advice. I know I probably wont be an astronaut when I grow up, but there is always a chance.

History:

Case 24:

In the 20th century, Presedant JFK landed the first man on the moon, Neil Armstrong. A company called NASA leads most outer space explorations. In sometime after that, the us a landed rovers on mars. Hopefully someday people can land there to. other countarys have landed on the moon to. but we were the first.

Journal Writing/Diary:

Case 2:

Astronaut Life: My Diary

Apr. 3/2028 “One small step for man, one large step for mankind”

So, whoever said this quote is so wrong! Guaranteed that they never walked on the moon or went to space… well maybe they did. I can tell you first hand that it is NOT one small step for me, seeing as I just got back from walking on the moon. Allow me to explain, just in case this ever gets published or something. It was recommended that we all write journals of our travels and, seeing as I didn’t want to disappoint, I followed the advice. So this is my journal of my journey in space. I guess writing help because it’s the easiest way to jot down fresh memories and reactions. I mean, we all know that brains can only hold so much! Well, it’s time for me to go have some freeze-dried ice cream. I'll write soon!

Apr. 7/2028 Wow! Has it already been 4 days! IT’s hard to keep track, since space always looks the same. Well we only stayed on the moon for a free days, seeing as it’s old, boring + has already been discovered. We just checked in there to get supplies. As you know, the new world peace center has been moved to he moon. Apparently the Earth couldn’t handle them or vice versa or something, at lease, that’s what I learned in school. So now we’re off to Mars. We’re actually pretty close + should be landing soon. I truly am bored up here. I brought magazines, knitting and loads of books… oh and plenty of freeze-dried ice cream! But still, it’s really lonely and space is just so vast. Ultimately, the mission is to test out light speed. But the problem is that if we try it too close to our solar system, we could disrupt the whole thing! So, we have to get far enough way first. How far is far enough? I have no clue! We'll we're almost there and I really should be going. But, I must say, I really like the writing thing. It gives me a nice continuum of at least doing something normal. Write all about Mars later.

Apr. 8/2028 So Mars was really awesome but boring too. How? Well, dear reader, let me tell you and then you can ask questions. I’m so glad Mars was the pitstop. I felt like I needed to stretch my legs. So it was very… red and rocky. Seeing as I’d been to the dessert in Salt Lake City sooo many time, it locked just like that! So if you want to see what Mars looks like, go to Salt Lake City.
